# A cross-sectional study of the relation between symptoms and physical findings in computer operators

**DOI:** 10.1186/1471-2377-6-40

**Published:** 2006-11-01

**Authors:** Jørgen R Jepsen, Gert Thomsen

**Affiliations:** 1 Department of Occupational Medicine, Ribe County Hospital, Østergade 81-83, DK-6700 Esbjerg, Denmark

## Abstract

**Background:**

The character of upper limb disorder in computer operators is subject to debate. A peripheral nerve-involvement is suggested from the common presence of a triad of symptoms consisting of pain, paraestesiae and subjective weakness, and from physical findings suggesting neuropathy. This study aimed to examine the outcome of a detailed neurological examination in computer operators and to compare findings with the presence of symptoms.

**Methods:**

96 graphical computer operators answered a modified Nordic Questionnaire including information on perceived pain in the shoulder, elbow, and wrist/hand scored for each region on a VAS-scale 0 – 9. In addition, they underwent a physical examination including the subjective assessment of the individual function of 11 upper limb muscles, of algesia in five and vibratory threshold in three territories, respectively, and of mechanosensitivity of nerves at seven locations. In order to reflect an involvement of the brachial plexus (chord level), the posterior interosseous nerve and the median nerve at elbow level we defined three patterns of neurological findings illustrating the course of nerves and their innervation. The pain scores summarized for the three upper limb regions (min. = 0, max = 27) in the mouse-operating and contralateral limbs were compared by a Wilcoxon test and the relation to each physical item analyzed by Kendall's rank correlation. The relation of summarized pain to each pattern was studied by application of a test of the trend across ordered groups (patterns).

**Results:**

Pain, paraestesiae and subjective weakness was reported for 67, 23, and 7 mouse-operating limbs, respectively, with the summarized pain scores exceeding 4 in 33 limbs. Abnormal physical findings were prevalent. The summarized pain was significantly related to a reduced function in five muscles, to mechanical allodynia at one location and to elevated threshold to vibration in two territories. Brachial plexopathy was diagnosed in 9/2, median neuropathy in 13/5 and posterior interosseous neuropathy in 13/8 mouse operating/contralateral limbs, respectively. The summarized pain was significantly higher in the mouse-operating limbs and in limbs with any of the defined patterns. There was a significant trend between the summarized pain and the summarized scores for the items contained in each pattern.

**Conclusion:**

This small-scale study of a group of computer-operators currently in work and with no or minor upper limb symptoms has indicated in symptomatic subjects the presence of peripheral nerve-afflictions with specific locations.

## Background

It has been estimated that two thirds of employees in the industrialized countries use a computer on a daily basis and that one out of five uses a computer at least 3/4 of the total work-time. In a recent study of computer operators working intensively with pointing devices, about 10.7% complained of moderate to severe pain in the neck and 7.7% in the right shoulder during the preceding week [1]. The occurrence of pain increased with computer work [1,2]. However, apart from a possible relation to tension neck, the physical examination in symptomatic workers was unable to indicate clinical somatic disorders responsible for the frequent and serious complaints [1,2].

The diagnostic difficulties pertaining to a proportion of work-related upper limb disorders including those related to the use of computers and pointing devices comprise patients with even severe upper limb pain. Consequently, these conditions are often characterized by non-specific diagnostic acronyms such as cumulative trauma disorder, repetitive strain injury or simply "mouse-arm". In addition to reflecting the diagnostic constraints this practice illustrates the need of a common understanding with regard to the type and location of the responsible pathology. Accordingly, there is also no consent as to an appropriate selection of physical tests which are likely to reflect complaints. Matters are further complicated by indications that somatization may act as a possible confounder or effect modifier in studies of occupational risk factors in non-specific arm pain [3].

In prior studies we have documented the reproducibility of a detailed semi-quantitative neurological examination of the upper limb nerves [4,5]. This examination included an assessment of the function of selected muscles while positioning the limb in order to favour a specific muscle and reduce the influence of others [4], of the presence of mechanical allodynia with pressure along nerve trunks, and of sensory deviations from normal in homonymously innervated territories [5]. The physical findings could be reliably integrated into patterns illustrating peripheral nerve afflictions with specific locations. The patterns were defined in accordance with the topography of the upper limb nerves and their muscular and sensory innervation [5]. The presence of patterns was related to the presence of symptoms [6].

This approach has been applied in 21 heavily exposed computer operators with severe upper limb complaints referred for assessment in a hospital department of occupational medicine. The neurological examination allowed the identification of a rather clear and consistent pattern suggesting a combined affliction of the brachial plexus at chord level and the posterior interosseous and median nerves on elbow level [7]. It is not known whether similar neurological patterns can be detected among computer operators in current occupation some of which may be symptomatic while the majority would be expected to be healthy.

On a sample of computer operators in active work we performed a neurological examination comprising muscle function, sensibility, and mechanosensitivity of nerve trunks in order to study the relation of pain to the mentioned physical findings and their occurrence in the defined patterns. We had a priori elaborated two hypotheses:

• The summarized pain score in the mouse-operating limbs exceeds the score in the non-mouse operating limbs;

• The summarized pain score is related to abnormal findings for the individual physical items contained in the three defined patterns and to the summarized scores for the three defined patterns.

## Methods

### Material

The study base consisted of 117 computer operators (engineers and technical assistants) in two divisions of the Danish engineering company Rambøll A/S. All computer operators were exposed to computer work for more than 20% of their total working time or had experienced upper limb symptoms within the last 12 months.

A sub-sample of 96 computer operators who answered a questionnaire about upper limb symptoms before the physical examination and accepted participation in the subsequent physical examination constituted the study group.

39 computer operators were female of median age 30 years (range 20–60) and 57 were male of median age 30 years (range 20–50). Their median body mass index was 24 (range 19–44) and 25 (range 21–33) for females and males, respectively, and their median professional computer experience was 124 months (range 13–492) and 101 months (range 17–307), respectively.

The study complied with the Helsinki declaration. It was approved by the local Ethics Committee (2487A-03) and signed informed consent was obtained from all participants.

### Questionnaire and physical examination

All 96 participants filled in a web based questionnaire which was based on the Nordic Questionnaire [8] and designed for electronic completion and submission. The posed questions included information about demographic data and pain experienced during the last three months in the shoulder, elbow, and hand/wrist on both sides. Each anatomical region was defined by drawings. The participants indicated their dexterity and the preferred hand for the pointing devise. Three used the pointing device with their left side and 11 with both hands. Two of these were left-handed and had their left side defined as the mouse operating limb. For the remaining 9 ambidextrous participants the right was assigned as operating the mouse. The respondents scored their perceived pain for each region on a VAS-scale 0–9. Subjective weakness and paraestesiae was registered but no further symptoms reported were used for this study.

Subsequently, the participants were subjected to a physical examination of selected neurological parameters (Table 1, 2, 3, 4) incorporating extracts of the examination protocol presented and validated previously [4,5].

The examiner was blinded to any patient-related information including the questionnaire data. No communication occurred during the physical examination except instructions from the examiner and reactions from the subject to the applied tests.

The following physical parameters were examined bilaterally according to methods formerly described in details [4,5]:

• The isometric motor function was manually assessed with 11 contractions each aiming to identify minor weaknesses in a specific muscle (Table 2) on a mere clinical basis. The inter-rater reliability of the testing method has been found moderate to good for most contractions [4] although the results were not validated relative to objective measurements. The assessment included the peak strength as well as the ability of the individual to hold the force at a constant level during testing thus containing a component of endurance. The assessment, however, could not distinguish between strength and endurance [4]. The two sides were examined simultaneously in order to reveal any discrepancy in the individual muscle function between the right and left side. If the examiner was in doubt with regard to presence or absence of weakness up to three repetitions were allowed for each contraction with no standardized rest period. The level of function for each contraction was graded between 0 and 5 with subdivision of grade 4 into 4-, 4, and 4+ [4,9] (Table 1). In order to stabilize the limb, minimize discomfort and ensure an optimal positioning during testing of a specific contraction while disfavouring the influence of others, specific postures were carefully defined to reflect each muscle [4]. The muscles were evaluated from proximal to distal with three standard postures of the upper limbs:

◦ The subject's arms were elevated horizontally forward, the elbows kept fully extended, the forearms pronated, the wrists kept at neutral and the hand clenched. Standing in front of the patient, the arm adduction (*pectoral*) and abduction (*posterior deltoid*) were tested by applying force against the subject's wrists from inward-out and from outward-in, respectively. The preferred exit position for the posterior deltoid is to have the subject keep the arms 30 degrees outward. The subject then lowered the arms with the elbows still fully extended but the forearms now in neutral and the clenched hands pressed toward the knees as the examiner was gripping the wrist and lifted the arms upward (*latissimus dorsi*);

◦ The subject's upper arms were now kept along the sides of the chest, the elbows flexed to right angle with the forearms pointing forward and kept in neutral position, the wrists kept in neutral and the hands clenched. Standing in front of the subject, the examiner leaned forward toward the subject's wrists, asking the patient to "carry" the examiner (elbow flexion, defined as *biceps*). Resisted by the subject the examiner then pressed the subject's clenched hands inward (*infraspinatus*). For this test, the subject's forearms were 30 degrees rotated in the outward direction. Finally, standing behind the subject, the examiner lifted the subject's wrists upward (*triceps*) against the subject's resistance;

◦ The subject leaned forward, resting the forearms on the thighs with the wrists just distal to the knees. With the subject's forearms fully supinated, hands clenched and the wrists slightly flexed, the examiner leaned forward, pressing toward the proximal interphalangeal joint knuckles of the index and long fingers to extend the wrists of the subject (*Radial flexor of wrist*). The subject then had the small fingers abducted and the examiner pressed at the tip of the fingers toward the ring finger (*Abductor of small finger*). The subject brought the thumbs into opposition and the examiner pressed them down toward the palm (*Short abductor of thumb*). With the subject's forearms fully pronated, hands open and wrists extended the examiner leaned forward, pressing against the knuckles of the index and long fingers to flex the subject's wrists (*Short radial extensor of wrist*). Finally, the distal part of the subject's forearm was firmly held by the examiner's one hand while pressing the ulnar-deviated wrist in the radial direction (*Ulnar extensor of wrist*).

• Algesia (needle prick) was assessed in five and the threshold to vibration by use of a tuning fork 256 Hz in three innervation territories (Table 3) as formerly described [5]. Deviation of sensibility was classified as "severely reduced/changed" when an allodynic reaction was recorded, or when pain or vibration could either not be perceived at all or was altered sufficiently to be clearly apparent to the examiner from the subject's reaction. Deviation of sensibility was classified as "reduced/changed" with any other divergence from normal (hypo- or hyper-sensibility). For the latter assessment, sensation was compared with sensibility in other territories assessed as normal (Table 1).

• The mechanosensitivity (soreness) of nerve trunks was assessed at seven locations by palpating with a moderate manual pressure of 3 kp from proximal to distal (Table 4). Mechanical allodynia was quantified according to Table 1. "Severe" mechanical allodynia was registered with avoidance reaction/jump sign, "moderate" allodynia when the subject expressed the pressure as seriously uncomfortable and "mild" allodynia with the presence of any other soreness exceeding normal. For the latter assessment, the level of mechanical allodynia was compared to reactions regarded as normal to pressure elsewhere along nerves (Table 1).

### Definition of peripheral nerve afflictions with specific locations

Clinical observations in patients with severe upper limb symptoms related to computer work has led to suggestions of three characteristic patterns of physical findings each of which reflecting a specific location of peripheral nerve affliction [7]. These patterns were defined in accordance with the topography of the upper limb nerves and their muscular and cutaneous innervation [5]. The employed criteria for each location were the following:

• Brachial plexus neuropathy, chord level: Weakness during the contractions reflecting the deltoid, biceps, and radial flexor of wrist muscles accompanied by increased threshold for vibration in the median nerve-territory, and soreness with a moderate pressure over the infraclavicular brachial plexus at chord level.

• Posterior interosseous nerve: Weakness during the contraction reflecting the ulnar extensor of wrist muscle and soreness with a moderate pressure over the posterior interosseous nerve at the arcade of Frohse.

• Median nerve, elbow level: Weakness during the contraction reflecting the radial flexor of wrist muscle and increased threshold of vibration in the median nerve-territory, and soreness with a moderate pressure over the median nerve at elbow level.

### Statistics

For each limb a new variable was created from the sum of scores for pain during the last three months in the shoulder, the elbow, as well as the hand and wrist. The resulting summarized score for pain in each limb would be in the range from 0 (no pain at all) to 27 (severe pain in all three regions).

The summarized pain scores in the mouse-operating and the contralateral limbs were compared by a Wilcoxon test.

In both the mouse-operating limbs and the contralateral limbs, the relation of the summarized pain scores to the scores for each contraction, for sensibility (algesia and vibration thresholds) in each territory, and for mechanosensitivity at each location were analysed by Kendall's rank correlation.

In addition, we applied a trend test across ordered groups, i.e. the summarized pain vs. the summarized scores for each of the patterns illustrating the three locations of neuropathy. The latter scores were developed by the addition of the scores for contraction reflecting each muscle, for sensibility (algesia and vibration thresholds) in each territory, and for mechanosensitivity at each location, respectively, as relevant to each pattern (Table 5).

The significance level was set to 0.05, but additionally adjusted for a potential mass significance by defining the level of significance according to the number of items examined (Bonferroni adjustment).

Data were processed by Stata ver. 8.2.

## Results

### Prevalence of symptoms

#### Pain

Pain during the last three months in the mouse-operating limb was reported by 67 out of the 96 study subjects and located to the shoulder in 42 subjects, to the elbow in 16 subjects, and to the hand or wrist in 40 subjects, respectively. The intensity of pain was mostly slight (median score = 2, range 0–16) with the summarized pain score exceeding 4 in 33 mouse operating limbs. For comparison, contralateral pain was experienced by 24. It was of minor intensity (median = 0, range 0–14) with a summarized score exceeding 4 in 13 limbs. There was a clear and highly significant difference between the summarized pain score in the mouse-operating vs. the contralateral limbs with pain being more severe in the former (z = 6.195, p < 0.0000).

#### Paraestesiae

Abnormal sensations such as numbness/tingling or paraestesiae in the mouse-operating/contralateral upper limb were experienced by 23/9 subjects, respectively.

#### Weakness

Subjectively reduced muscle weakness or abnormal fatigability in the mouse-operating/contralateral limb was experienced by 7/6 subjects, respectively.

Five subjects complained of pain, paraestesiae, and weakness in the mouse-operating limbs while one had this combination on the contralateral side.

### Prevalence of physical findings

#### Individual findings

Physical findings assessed as abnormal by the examiner were prevalent. Weakness was found in 56 mouse operating and 25 non-mouse operating limbs (Table 2). Abnormalities with regard to algesia and threshold to vibration in any examined cutaneous innervation territories were identified in 40/9 and 66/14 mouse operating/non-mouse operating limbs, respectively (Table 3). Mechanical allodynia of nerve trunks at any of the studied locations was present in 60/34 mouse operating/non-mouse operating limbs (Table 4).

#### Patterns of findings

**Figure 1:**
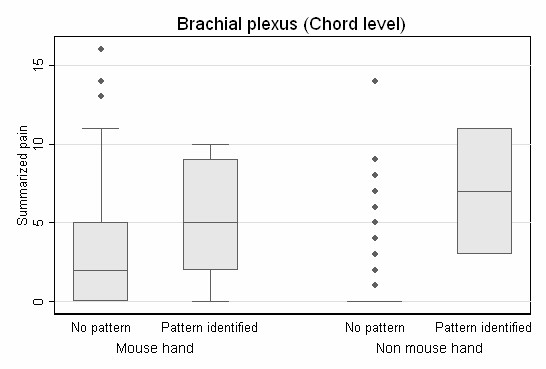
**Box plots illustrating the median summarized pain score with brachial plexus affliction at chord level**. Findings in the mouse operating versus the non-mouse operating limb. The median for each dataset is indicated by the line in the boxes which in themselves contain 50% of the data. The upper and lower hinges of the boxes indicate the 25–75 percentiles. Values within 1.5 times the interquartil range are indicated by the lines. The individually plotted points at a greater distance from the median represent outliers.

**Figure 2:**
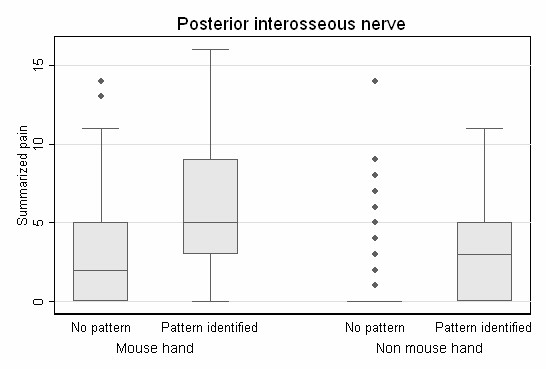
**Box plots illustrating the median summarized pain score with posterior interosseous nerve affliction at elbow level**. Findings in the mouse operating versus the non-mouse operating limb. The median for each dataset is indicated by the line in the boxes which in themselves contain 50% of the data. The upper and lower hinges of the boxes indicate the 25–75 percentiles. Values within 1.5 times the interquartil range are indicated by the lines. The individually plotted points at a greater distance from the median represent outliers.

**Figure 3:**
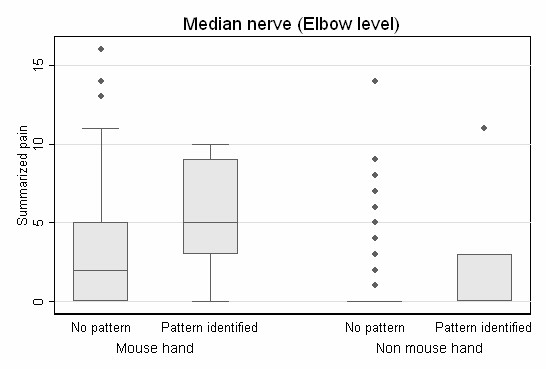
**Box plots illustrating the median summarized pain score with median nerve affliction at elbow level**. Findings in the mouse operating versus the non-mouse operating limb. The median for each dataset is indicated by the line in the boxes which in themselves contain 50% of the data. The upper and lower hinges of the boxes indicate the 25–75 percentiles. Values within 1.5 times the interquartil range are indicated by the lines. The individually plotted points at a greater distance from the median represent outliers.

The simultaneous presence of reduced muscle function, sensory deviations from normal and mechanical allodynia allowed the classification into the patterns defined to reflect specific syndromes involving the peripheral nerves: Whether in accordance with an involvement of the brachial plexus at chord level, the posterior interosseous nerve or the median nerve at elbow level, the patterns were much more frequently identified on the mouse operating side than on the contralateral side. Out of 96 patients, 9/2 mouse operating and non-mouse operating limbs satisfied the criteria for an affliction of the brachial plexus at chord level, 14/8 for posterior interosseous neuropathy, and 13/5 for median neuropathy at elbow level, respectively (Figures 1, 2, 3). Patterns reflecting neuropathy at all these locations were present in 7/2 subjects, while a pattern of neuropathy in at least one location was present in 17/9 subjects, respectively.

### Correlation between symptoms and physical findings

With the level of significance set to 0.05, the summarized pain correlated significantly to the functional level of contractions reflecting the muscles that could be involved with neuropathy at the three locations (deltoid, biceps, radial flexor of the wrist, short radial extensor of wrist, and ulnar extensor of the wrist). There was a borderline but non significant relation to the function of contractions reflecting the triceps and short abductor of thumb muscles. For the pectoral, latissimus and infraspinatus muscles and the small abductor of the fifth digit muscle there was no relation to pain (Table 2). For sensibility, the only significant correlations to summarized pain were identified for elevated threshold to vibratory stimulation in the median and radial nerve territories (Table 3). Significant abnormalities regarding mechanical allodynia were found for the posterior interosseous nerve (Table 4). Several other relations did not reach significance (Table 3 and 4).

After Bonferroni adjustment, the function of the short radial extensor of wrist muscle, the vibratory threshold for the median nerve territory and the mechanosensitivity of the posterior interosseous nerve remained significantly related to the summarized pain (Table 3).

The application of a trend test across ordered groups resulted in a significant trend between the summarized pain score and classification as a nerve affliction on each of the three locations on both sides. Significance remained for afflictions of the brachial plexus and median nerve after Bonferroni adjustment (Table 5).

Comparable relations were found on the contralateral side (Table 5).

## Discussion

Conventionally, regional physical findings have been compared to pain in the same region. It is, however, well appreciated that the pathology in upper limb conditions may be situated at a distance to the dominant location of pain. We have deliberately established the dependent variable as a measure of integrated upper limb pain by adding the scores from each of three regions. In this rather small sample of computer operators this summary measure has indicated less and minor upper limb pain than reported elsewhere [1,2,10,11]. On this background, the relations between pain and findings are particularly noteworthy. The application of a trend test across ordered groups justified by the gradual transition from "normal" to "abnormal" of the examined physical parameters resulted in a significant correlation between perceived pain and physical findings suggestive of afflictions of the three locations of nerve affliction hypothesized as characteristic for computer-related upper limb disorder [7]: The brachial plexus at chord level, the posterior interosseous nerve at the Arcade of Frohse and the median nerve at elbow level. The trends were present in the mouse operating as well as in the non-mouse operating limb (Table 5). However, symptoms and findings were much more frequent in the former.

After application of the Bonferroni correction the significant relations to pain (Table 2, 3, 4, 5) were preserved for several items (muscle function, threshold for perception of vibration and nerve-mechanosensitivity) and their occurrence in patterns. Except for contractions reflecting the pectoral and latissimus muscles on the non-mouse operating side and the infraspinatus muscle on the mouse operating side all relations had the same direction. This observation indicates that relations showing significance before Bonferroni correction cannot be due to mass significance [12]

### Selective nerve-affliction influencing the function of specific contractions while other contractions were intact

Pain correlated to muscle weaknesses compatible with nerve afflictions at the three specific locations but was not related to the function of muscles that did not contribute to the patterns (Table 2). This can be explained by anatomical facts: The suprascapular and thoracodorsal nerves depart from the upper trunk and from the divisions, respectively, i.e. proximally to a brachial plexus affliction at chord level [7]. Accordingly, the function of the infraspinatus and latissimus dorsi muscles should be intact unless further spread within the brachial plexus has occurred, and the summarized pain should not differ between limbs with intact and reduced function in these two muscles. In analogy, a pectoral and an ulnar nerve affliction would be less likely. The pectoral nerve is located in the vicinity of the medial chord which forms the ulnar nerve. Compared to the narrow surroundings of the lateral and posterior chord, the available space around the medial chord favours mobility of this part of the brachial plexus. The rare occurrence of an ulnar nerve affliction at elbow and hand level [7] was confirmed by a comparable level of pain in limbs with intact function and limbs with reduced function in the abductor of small finger muscle (Table 2). The findings relating to the sensibility and mechanosensitivity of nerves were concurrent with these observations.

### Positive findings in the absence of significant pain

Slight degrees of muscle weakness, hypalgesia, increased threshold to vibratory stimulation, and mechanical allodynia along the course of nerves were found in a high proportion of the participants out of which most had minimal or no upper limb complaints. This might question the validity of the physical examination which, however, has been previously studied in a sample of patients some of which with upper limb disorder of a more severe character. There was a good inter-observer reliability. The two blinded examiners reached a high correlation (95% CI) between their findings including the patterns defined for the brachial plexus at chord level 0.82 (0.73–0.88), for the posterior interosseous nerve 0.75 (0.64–0.83), and for the median nerve at elbow level 0.82 (0.73–0.88), respectively [5]. With agreement between the two examiners, the pre-test odds for a limb to be symptomatic amounted to 0.46 and the post-test probability to 0.81. For each examiner the post-test probability was 0.87 and 0.88, respectively. These findings suggest the presence of construct validity of the physical examination [6].

Another interpretation of positive findings in the absence of pain would be that the physical examination is highly sensitive and that the outcome relates not only to a symptomatic disorder but potentially also to a pre-clinical minor dysfunction which may possibly be related to the exposure. The ability to predict pain from the defined patterns of findings (rather than findings in isolation) is an indication of the diagnostic potential in terms of identifying clinical cases of computer-related upper limb disorder and may suggest the applicability of the examination for screening purposes.

### Bilateralism of findings

The clear difference between symptoms and findings in the mouse operating and contralateral limb does provide some further validation to our findings. Still, however, it is noteworthy that similar neurological findings and patterns were observed at the two sides. Since the early descriptions on scriveners by Ramazzini [13] the tendency to spread of work-related upper limb pain to the contralateral limb has been acknowledged by many clinicians. This phenomenon may be due to factors such as substitution by work being taken over by the intact limb or to central processing of sensory inputs. With computer work the identical exposure from keyboarding with both hands may also influence.

### Subjective data

Although the relation between upper limb pain and nerve afflictions with characteristic locations is supported by this study it can be argued that the data are of a subjective or semi-subjective character and that results may accordingly be biased by subjectivity of the examiner as well as the examinee. Such bias may influence research based on clinical parameters as in the current study but also clinical work in which the anamnestic information and most physical qualities, e.g. those in the neurological examination, are of a subjective character. Still, in the absence of better or more practical measures we have to rely on clinical information with this limitation.

#### Subjectivity of the examiner

In order to reduce subjectivity all physical assessments and the interpretation of findings were performed blinded to any information about the studied computer operators. Still, it cannot be excluded that one finding, e.g. of weakness with a specific contraction, can bias other findings such as sensory deviations because all physical examinations were made by the same examiner.

#### Subjectivity of the examinee

Cross-sectional data suggest that psychosocial factors or somatization may explain non-specific arm pain in workers [3] and such mechanisms have also been proposed for computer operators [14]. Currently, there is no indication that a reduced threshold to the perception of pain is responsible [15]. In this study of a mostly healthy group of computer operators we regard an influence of psychological factors as unlikely for a number of reasons:

• Neurological "abnormalities" were frequent in non-symptomatic subjects, e.g. we found reduced muscle function in 56 mouse operating limbs out of which 13 were completely without pain and only 7 had subjective weakness. Reduced muscle function in the absence of subjective weakness is a common finding in upper limb patients including computer operators [7]. We have noticed at many occasions the surprise of patients with no complaints relating to muscular contraction when during testing they clearly perceive a weakness of which they were previously unaware.

• When asked to simultaneously do their best on both sides the examined subjects would not be likely to deliberately exert less force than they are capable to. With an actual identical sensibility the examined subjects would also hardly report a different perceived sensation in two compared territories. For mechanosensitivity, the presence of a more severe allodynia is readily visible from the reaction of the subject. It should be remembered that this study deals with generally healthy subjects who are actively working in an attractive enterprise. Malingering would not provide them any additional benefit.

• The selectivity of neurological findings, e.g. a weakness tending to affect certain muscles with others remaining normal and the rarity of nerve-afflictions elsewhere than at the three locations mentioned, also arguments against a major role of a psychological reaction. Most importantly, however, for the examinees to construct the patterns would demand a familiarity with the innervation and topography of nerves which is not plausible.

### Previous findings

In a smaller case study of 21 computer operators with severe upper limb symptoms [7], we have previously found a differential involvement of the examined muscles with a generally reduced function in contractions reflecting the deltoid, biceps, triceps, radial flexor of wrist, short radial extensor of wrist, and ulnar extensor of wrist muscles while other muscles remain intact. Together with sensory deviation from normal and mechanical allodynia of nerve trunks, characteristic patterns were identified. The current study supports the raised hypotheses by demonstrating comparable but minor findings in computer operators in work with relatively fewer symptoms. Findings are also comparable to those of Pascarelli et al. who studied 485 upper limb patients out of which 70% were computer operators. A neurogenic thoracic outlet syndrome in 70% was suggested by tests stressing the brachial plexus and by the demonstration of localized mechanical allodynia [16].

Jensen et al. found normal or only slightly reduced strength in symptomatic computer users compared to non-symptomatic computer users and controls [17]. The apparent divergence to our findings may be a question of statistical power or could be explained by the way the muscle function was assessed. In contrast to the attempt in the current study to manually assess the individual muscle function, Jensen et al. performed instrumental measurements of the integrated strength of several muscles. In addition, we have deliberately aimed to fatigue the subjects during testing by allowing up to three reiterations of each test [4].

We consider a detraining effect a less likely explanation for the identified weaknesses. The mostly minor level of perceived pain and the completely normal level of functioning would not cause symptomatic subjects to protect themselves by sparing their upper limb muscles and eventually loose strength. Another argument against a detraining effect is the selective weakness involving certain muscles while the function of others remained normal.

In a recent cross-sectional study of almost 7000 computer operators the physical examination disclosed a limited number of clinical upper limb disorders, similar to what would be expected in the general population [1,2]. Numbness or tingling was reported by 10.9%. Symptoms located to the median nerve territory in 4.8% and causing nocturnal symptoms in 1.4% could potentially be attributed to carpal tunnel syndrome but were unexplained in the remaining subjects [18]. Defined by localized palpation tenderness with withdrawal and pain with provocative manoeuvres, 12 prevalent nerve entrapments were diagnosed (supinator and pronator syndromes) and no new cases were identified at follow up after one year. A detailed neurological assessment was not included in the physical examination [19]. The perception of vibration was reduced in a small sub-sample of the same material consisting of subjects with paresthesia but without pain [14].

In a study of 533 visual display terminal workers upper limb disorders in 22% were dominated by tendon and muscle related conditions in 15% and 8%, respectively, and probable nerve entrapment in 4% [10]. Among 632 newly hired computer operators, the one year incidence of neck and shoulder symptoms was 58% and of hand/arm symptoms 39%, respectively. Symptoms were predominantly explained by "somatic shoulder/neck syndrome" and de Quervain's syndrome [11]. However, diagnoses depend on the selection and validity of the applied clinical tests and diagnostic criteria. Accordingly, the two diagnostic categorizations covering almost all symptomatic cases in the latter study can be questioned. "Somatic shoulder/neck syndrome" was characterized by non-specific signs and an absence of well-defined pathology. A positive Finkelstein test is neither pathognomonic nor specific for de Quervain's syndrome. Both may well represent a neuropathic condition.

### Non-specific upper limb pain

Increasing evidence is linking peripheral nerve dysfunction to conditions with "non-specific" upper limb pain: While some studies found similar nerve conduction velocity and vibrotactile perception in healthy computer users and non-exposed controls [20] others have demonstrated sub-clinical median nerve impairment [21] and elevated threshold for perception of vibration [17,22] persisting along with symptoms [23]. A recent study demonstrated a 15% increased threshold for vibration in computer operators with paraestesiae in contrast to those without paraestesiae [14].

Other indices of nerve-involvement in similar patients include abnormal reactions to upper limb tension tests [24], reduced nerve mobility [25], mechanical allodynia with slight pressure over nerve trunks [26], changed axonal flare reaction [27], secondary hyperalgesia [28], allodynic response to supra-threshold vibratory stimulation [22], and sympathetic reflexes [29]. Altered motor control [30], recruitment pattern [31] and movement strategies [32] in symptomatic office workers may also reflect an upper limb nerve-involvement. Combined with clinical experiences these findings may represent reactions to lesions or loading of peripheral nerves [15,33-37] and suggest the involvement of multiple nerve entrapment in "non-specific" upper limb conditions including those occurring in office employees [16,38,39].

## Conclusion

This cross-sectional study of computer operators has identified individual and patterns of neurological findings reflecting the upper limb peripheral nerves. The relation to upper limb pain of the presence of three specific patterns suggests a nerve involvement at explicit locations: The brachial plexus at chord level and the posterior interosseous and median nerves at elbow level. The present findings seem to confirm hypotheses with regard to the character and location of computer-related upper limb disorder emanating from clinical observations and an attempted pathophysiological explanation.

## Competing interests

The author(s) declare that they have no competing interests.

## Authors' contributions

GT and JRJ have designed the study and collected the data. GT has been responsible for data-management and both authors performed the statistical calculations. JRJ has been responsible for the preparation of the manuscript which has been approved by GT.

## Pre-publication history

The pre-publication history for this paper can be accessed here:



## Figures and Tables

**Table 1:** Quantification of the neurological qualities examined [4,5,9].

**Examined quality**	**Interpretation**
Manual testing of individual muscles	5 Contraction against powerful resistance/normal function = 0 4+ Contraction against gravity and strong resistance = 1 4 Contraction against gravity and moderate resistance = 2 4- Contraction against gravity and slight resistance = 3
Mechanosensitivity with slight pressure along nerve trunks	No soreness = 0 Mild mechanical allodynia = 1 Moderate mechanical allodynia = 2 Severe mechanical allodynia = 3
Sensibility examined by needle prick (algesia) and tuning fork 256 Hz (vibratory threshold)	Normal sensibility = 0 Reduced/changed sensibility = 1 Severely reduced/changed sensibility = 2

**Table 2:** Analysis by Kendall's rank correlation of the relation between contractions reflecting individual muscle function and the summarized pain in 96 participants

**Muscle**	**Number of limbs with weakness (median pain score)**							
					
	**Mouse operating limbs**		**Non-mouse operating limbs**		**Mouse operating limbs**		**Non-mouse operating limbs**	
	
	**Grade 4+**	**Grade 4 or less**	**Grade 4+**	**Grade 4 or less**	**Kendall's tau-b**	**P**	**Kendall's tau-b**	**P**
Pectoral	1 (3)	-	1 (0)	1 (0)	0.018	0.8544	-0.0553	0.5852
Deltoid	17 (3)	3 (5)	12 (1 1/2)	-	0.1731	0.0498	0.2092	0.0316
Latissimus	6 (5 1/2)	-	1 (0)	-	0.1023	0.2546	-0.0553	0.5852
Infraspinatus	9 (2)	-	13 (0)	-	-0.0439	0.6271	0.1100	0.2599
Biceps	14 (5 1/2)	2 (3 1/2)	7 (5)	-	0.1920	0.0303	0.3740	0.0001
Triceps	37 (3)	1 (5)	16 (0)	-	0.1500	0.0918	0.1969	0.0430
Radial flexor of wrist	18 (4)	2 (5)	8 (3)	-	0.1934	0.0297	0.2444	0.0120
Short radial extensor of wrist	18 (6)	2 (5)	13 (3)	-	0.2597	0.0033	0.2637	0.0067
Ulnar extensor of wrist	26 (5)	3 (4)	11 (3)	-	0.2045	0.0204	0.2348	0.0158
Short abductor of thumb	27 (3)	1 (5)	10 (4)	1 (11)	0.1475	0.0975	0.4044	0.0000
Abductor of small finger	6 (3)	-	3 (5)	-	0.0117	0.9020	0.2794	0.0041

The frequency and severity (as rated in Table 1) of muscle weakness is additionally illustrated. Significance level 0.05. Bonferroni adjustment 0.05/11 = 0.005

**Table 3:** Analysis by Kendall's rank correlation of the relation between algesia and vibratory threshold in individually innervated territories and the summarized pain in 96 participants

**Sensory innervation territory**		**Number of limbs with sensory abnormalities (median pain score)**							
						
		**Mouse operating limbs**		**Non-mouse operating limbs**		**Mouse operating limbs**		**Non-mouse operating limbs**	
		
		**Slight**	**Severe**	**Slight**	**Severe**	**Kendall's tau-b**	**P**	**Kendall's tau-b**	**P**
**Algesia**	Axillary	29 (4)	-	8 (0)	-	0.1557	0.0817	0.0719	0.4640
	Musculocutaneous	23 (4)	1 (5)	5 (0)	-	0.1507	0.0905	0.0863	0.3797
	Median	16 (4 1/2)	-	1 (3)	-	0.1053	0.2400	0.1404	0.1545
	Radial	22 (4 1/2)	-	3 (0)	-	0.1871	0.3640	0.0175	0.8680
	Ulnar	1 (8)	-	1 (0)	-	0.1097	0.2258	-0.0553	0.5852

**Vibration**	Median	56 (3 1/2)	2 (2 1/2)	14 (0)	-	0.2527	0.0043	0.1192	0.2217
	Radial	56 (3 1/2)	-	11 (0)	-	0.2108	0.0183	0.0169	0.8678
	Ulnar	22 (3 1/2)	-	6 (0)	-	0.1214	0.1749	0.0560	0.5704

The frequency and severity of sensory (as rated in Table 1) deviations from normal is additionally illustrated. Significance level 0.05. Bonferroni adjustment 0.05/8 = 0.006

**Table 4:** Analysis by Kendall's rank correlation of the relation between mechanical allodynia over nerves and the summarized pain in 96 participants

**Mechanosensitivity of nerve trunks**	**Number of limbs with mechanical allodynia (median pain score)**							
					
	**Mouse operating limbs**		**Non-mouse operating limbs**		**Mouse operating limbs**		**Non-mouse operating limbs**	
	
	**Mild**	**Moderate to severe**	**Mild**	**Moderate to severe**	**Kendall's tau-b**	**P**	**Kendall's tau-b**	**P**
Supraclavicular brachial plexus	6 (5)	-	-	1 (8)	0.0968	0.2812	0.1681	0.0875
Clavicular brachial plexus	4 (7)	1 (0)	-	-	0.1296	0.1469	-	-
Infraclavicular brachial plexus	23 (4)	11 (5)	13 (0)	2 (8)	0.1442	0.0947	0.1121	0.2463
Median nerve (elbow)	24 (3)	7 (5)	16 (0)	2 (8)	0.1110	0.2027	0.2187	0.0257
Posterior interosseous nerve	37 (4)	9 (8)	26 (0)	1 (5)	0.2768	0.0013	0.2463	0.0109
Ulnar nerve (sulcus)	-	-	1 (14)	-	-	-	-	-
Median nerve (carpal tunnel)	-	-	1 (11)	-	-	-	-	-

The frequency and severity (as rated in Table 1) of localized mechanical allodynia is additionally illustrated. Significance level 0.05. Bonferroni adjustment 0.05/7 = 0.007

**Table 5:** Test of trend across ordered groups of localized neuropathy and summarized pain in 96 participants

**Location of neuropathy**	**Items contained in the model**			**Mouse operating limbs**		**Non-mouse operating limbs**	
	
	**Muscles**	**Locations for mechanical allodynia**	**Algesia in sensory territories**	**Z**	**P**	**Z**	**P**
Brachial plexus (chord level)	Deltoid, biceps, radial flexor of wrist,	Infraclavicular brachial plexus	Median	2.77	0.006	2.91	0.004
Posterior interosseous nerve	Ulnar extensor of wrist	Posterior interosseous nerve	-	2.20	0.028	2.62	0.009
Median nerve (elbow level)	Radial flexor of wrist, short abductor of thumb	Median nerve (elbow)	Median	2.70	0.007	2.43	0.015

Significance level 0.05. Bonferroni adjustment 0.05/3 = 0.017
